# High dose antipsychotic therapy (HDAT) prescibing practice within the south trafford community mental health team

**DOI:** 10.1192/bjo.2021.313

**Published:** 2021-06-18

**Authors:** Oli Sparasci, Luis Rojo

**Affiliations:** 1 Greater Manchester Mental Health NHS Foundation Trust, The University of Manchester; 2 Greater Manchester Mental Health NHS Foundation Trust

## Abstract

**Aims:**

High Dose Antipsychotic Therapy (HDAT) is defined by the Royal College of Psychiatrists as either: “A total daily dose of a single antipsychotic which exceeds the upper limit stated in the BNF” or “A total daily dose of two or more antipsychotics which exceeds the BNF maximum as calculated by percentage.”

The use of HDAT is associated with significant risks to physical health and as such requires regular monitoring of various physiological parameters such as ECG, bloods and an assessment of cardiometabolic risk.

Following previous audits of HDAT prescribing practice in the inpatient setting within Greater Manchester Mental Health (GMMH) NHS FT, an audit of HDAT prescription in a general adult CMHT was conducted in Summer 2020, with the following aims:

To identify patients in the South Trafford CMHT who are prescribed HDAT.

To assess the prescription of HDAT against local guidance on the use of unlicensed medications.

To highlight good practice and areas for improvement in the prescription of HDAT.

**Method:**

All patients under the South Trafford CMHT in Summer 2020 were identified. Current prescriptions for antipsychotic medication were ascertained through review of electronic patient records. Those noted to be on HDAT were assessed against audit criteria derived from the GMMH Unlicensed Medicines Policy, previous audits of HDAT use and the RCPsych consensus report on HDAT prescription.

**Result:**

11 of 252 patients (4%) were identified as being on HDAT, of which eight were due to polypharmacy and three to high dose of a single antipsychotic. For 1/11 patients target symptoms and a risk/benefit rationale were documented. The mean length of time on HDAT was 6 years. 7/11 patients had either tried or considered clozapine in the past. 8/11 patients had not had an ECG within the last year, 4/11 had not had yearly U&E. 8/11 had regular mental health reviews.

**Conclusion:**

Compliance with the audit standards was found to be highly variable. This may reflect many factors, including the length of time since commencing HDAT and the complex shared care arrangements currently in place in Trafford. Thus, the following recommendations have been made:

To start a register of all patients prescribed HDAT.

To review local guidelines and documentation to ensure they are up to date and can be effectively implemented in routine clinical practice.

To ensure that the responsibility for conducting yearly physical health checks for patients prescribed HDAT is communicated to the relevant parties.

